# Group empathy for pain is stronger than individual empathy for pain in the auditory modality

**DOI:** 10.1093/scan/nsae074

**Published:** 2024-10-17

**Authors:** Min Shao, Yulan Qiu, Yudie Zhang, Huiling Qian, Zilong Wei, Mingyu Hong, Shuqin Liu, Jing Meng

**Affiliations:** Research Center for Brain and Cognitive Science, Chongqing Normal University, Chongqing 401331, China; Key Laboratory of Applied Psychology, Chongqing Normal University, Chongqing 401331, China; Research Center for Brain and Cognitive Science, Chongqing Normal University, Chongqing 401331, China; Key Laboratory of Applied Psychology, Chongqing Normal University, Chongqing 401331, China; Research Center for Brain and Cognitive Science, Chongqing Normal University, Chongqing 401331, China; Key Laboratory of Applied Psychology, Chongqing Normal University, Chongqing 401331, China; Research Center for Brain and Cognitive Science, Chongqing Normal University, Chongqing 401331, China; Key Laboratory of Applied Psychology, Chongqing Normal University, Chongqing 401331, China; Research Center for Brain and Cognitive Science, Chongqing Normal University, Chongqing 401331, China; Key Laboratory of Applied Psychology, Chongqing Normal University, Chongqing 401331, China; Research Center for Brain and Cognitive Science, Chongqing Normal University, Chongqing 401331, China; Key Laboratory of Applied Psychology, Chongqing Normal University, Chongqing 401331, China; Research Center for Brain and Cognitive Science, Chongqing Normal University, Chongqing 401331, China; Key Laboratory of Applied Psychology, Chongqing Normal University, Chongqing 401331, China; Research Center for Brain and Cognitive Science, Chongqing Normal University, Chongqing 401331, China; Key Laboratory of Applied Psychology, Chongqing Normal University, Chongqing 401331, China

**Keywords:** empathy for pain, pain, group, auditory, ERP

## Abstract

Humans live in collective groups and are highly sensitive to perceived emotions of a group, including the pain of a group. However, previous research on empathy for pain mainly focused on the suffering of a single individual (“individual empathy for pain”), with limited understanding of empathy for pain to a group (“group empathy for pain”). Thus, the present study aimed to investigate the cognitive neural mechanisms of group empathy for pain in the auditory modality. The study produced group painful voices to simulate the painful voices made by a group, and recruited 34 participants to explore differences between their responses to group painful voices and individual painful voices using the event-related potential (ERP) techniques. The results revealed that group painful voices were rated with higher pain intensity, more negative affective valence, and larger P2 amplitudes than individual painful voices. Furthermore, trait affective empathy scores of the participants were positively correlated with their P2 amplitudes of group painful voices. The results suggested that the group empathy for pain may facilitate affective empathetic processing in auditory modality.

## Introduction

Empathy for others’ emotions is essential in the social interactions (Singer et al. 2004, Smith et al. 2007). From a young age, children begin to perceive emotions of groups (Powell and Spelke 2013), enabling them to quickly empathize with the groups. The empathetic ability to understand emotions of an individual or a group is critical of successful social interaction (Singer et al. 2004, Smith et al. 2007), and the ability to perceive and judge emotions of a group (i.e. group empathy) may be even more evolutionarily significant than empathy for a single individual (i.e. individual empathy). Most emotions experienced by individuals are generated within social contexts (Smith et al. 2007). When individuals identify with a group, the group becomes part of their self-concept and acquires social and emotional significance (Smith and Henry 1996). Consequently, emotions can elicit by group-related events (Smith et al. 2007). Individuals may feel guilty for events that occurred in their country long before they were born (Doosje et al. 1998). Furthermore, even on an individual level, people can experience emotions aligned with group identities (Smith et al. 2007). In real life, people also often encounter situations in which multiple individuals express emotions at the same time (Mei et al. 2024). Thus, group empathy can enhance individuals’ ability to perceive group emotions and facilitate their integration into society. However, previous research in empathy mainly focuses on individual empathy, with limited attention to group empathy. The ability to perceive and judge the pain of a group (i.e. group empathy for pain), a topic on which few studies have been conducted. Therefore, the present study explored the difference between group empathy for pain and individual empathy for pain and cognitive neural mechanism in auditory modality.

Empathy for pain, as a particular form of empathy, includes cognitive processing such as identification and judgment of others’ pain (cognitive empathy for pain) as well as emotional resonance and emotional response to others’ pain (affective empathy for pain; Singer et al. 2004, Fan et al. 2011, Ren et al. 2022). A deeper understanding of the mechanisms of both group and individual empathy for pain is of significant value for comprehending interpersonal interactions, emotional communication, and the functions of empathy (Meng et al. 2013, Peng et al. 2021). Individual empathy for pain can be experienced through multiple sensory modalities (Meng et al. 2017), with previous research mainly focusing on the visual and auditory modalities. In the visual experiments, researchers typically use images or videos of an actor’s painful hand or foot (Fan and Han 2008, Fan et al. 2011, Meng et al. 2013, Peng et al. 2019, Li et al. 2023, Wei et al. 2023), painful facial expression (Cheng et al. 2007, Li et al. 2020), or painful action (Li et al. 2022) as experimental stimuli. In the auditory experiments, researchers generally use recordings of individual painful voices, such as the recordings of “ah” voice expressing pain made by an actor (Meng et al. 2019a, 2020) as experimental stimuli. However, auditory stimuli related to others’ pain typically convey more emotional information compared to visual stimuli (Meng et al. 2019a, Shao et al. 2024). Additionally, auditory stimulation can better aggregate group emotions simultaneously and mitigate the position effect that can arise from the presentation of visual group information. Therefore, using auditory stimulation in group empathy research may be a more effective choice. The ERP studies of empathy for pain in the auditory modality (Liu et al. 2019, Meng et al. 2019a, 2020, Shao et al. 2024) had found individual painful voices elicited N1, P2, and late negative components (LNCs). N1 and P2 represented early affective empathy (Choi et al. 2014), while LNC represented late cognitive empathy (Meng et al. 2019a, 2019b, Shao et al. 2024).

Previous research on empathy related to group emotions mainly focused on how the surrounding group emotional information affected the empathetic responses to the target individual (Wieser and Brosch 2012, Gray et al. 2017, Griffiths et al. 2018, Cao et al. 2023, Wang et al. 2024). For example, a previous study (Mumenthaler et al. 2018) simultaneously presented a target face surrounding with another emotional face and asked participants to judge the emotion of the target face. The results revealed that when the emotions of these faces were congruent, participants made quicker and more accurate responses in recognizing the target face’s emotion compared to when the emotions were incongruent (Mumenthaler et al. 2018). The previous research on empathy and intra-racial prejudice found that viewing painful expressions of individuals from the same race elicited larger P2 and N2 amplitudes compared to viewing neutral expressions from the same race (Sheng and Han 2012, Sheng et al. 2014). So far, only one previous study explored group empathy for pain in the visual modality (Mei et al. 2024), they found that compared to one painful face, simultaneous presentation of four painful faces elicited enhanced empathetic responses, including higher pain intensity scores, more negative emotional reactions, and larger P2 amplitudes. Moreover, previous studies have indicated that the P2 component is modulated not only by emotional quality (Paulmann et al. 2009, Yeh et al. 2016) but also by contextual information (van Wassenhove et al. 2005). Thus, these results suggest that group-related effects may occur early in empathy.

To address the aforementioned issues, the present study aimed to produce group painful voices and utilized behavioral and ERP techniques to investigate the cognitive neural mechanisms of group empathy for pain. Based on previous studies (Sheng and Han 2012, Sheng et al. 2014, Meng et al. 2019a, Mei et al. 2024, Shao et al. 2024), the present study hypothesizes that: participants will exhibit stronger empathetic behavioral responses and enhanced ERP amplitudes associated with affective empathy for pain to group painful voices than individual painful voices.

## Methods

### Participants

An a priori power analysis using G*Power 3.1 (Faul et al. 2009) indicated that a sample size of 30 participants was needed to attain a statistical power of 0.9 for detecting median-sized effects (*f* = 0.25) with an alpha level of 0.05 in a two-factor within-participants repeated measures analysis of variance (ANOVA). Taking into account the possibility of dropouts or errors during the study, a total of 34 participants (16 females; age = 20.44  ± 1.52 years) were recruited for this study. All participants were right-handed, had normal or corrected-to-normal vision, normal hearing, no history of neurological or psychiatric disorders, and were not currently using any medication. Prior to the experiment, all participants were briefed on the experimental procedures and signed informed consent forms. The study was approved by the Chongqing Normal University Academic Ethics Committee, and all procedures were performed in accordance with the ethical guidelines and regulations.

### Stimuli

From the Empathy for Pain Stimuli System (EPSS; Meng et al. 2024), 20 painful voices from 20 actors (10 females) and 20 matched nonpainful voices were selected to serve as individual painful and nonpainful voices, which are “ah” voices made by 20 actors. The individual painful voices express the pain feelings experienced by the actors, whereas the individual nonpainful voices are neutral voices that do not convey any negative or positive feelings. The duration of individual painful and nonpainful voices is 1000 ms, with matched average loudness.

**Figure 1.**
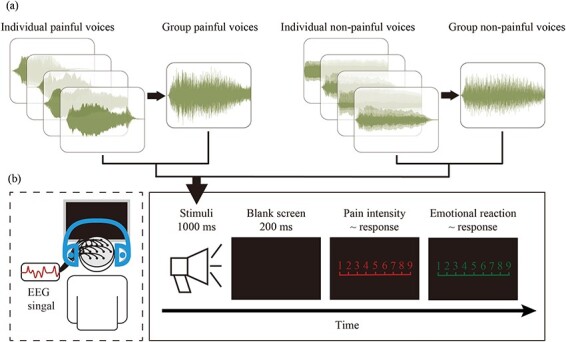
Flowchart describing the stimuli examples, experimental design, and procedure. (a) The examples of waveforms of group and individual painful and nonpainful voices. Each group painful and nonpainful voice was made by superimposing four randomly selected individual painful and nonpainful voices, respectively. (b) Experimental design and procedure. EEG signals were simultaneously recorded while participants performed the experimental task.

To make group voices, Adobe Audition 2022 software (Adobe Systems, Inc.) was used to superimpose the individual painful voices and individual nonpainful voices into group painful and nonpainful voices, respectively, as shown in Fig. 1a. For group painful voices, four individual painful voices were randomly selected and superimposed to create a group painful voice, simulating the voices made when a group experienced pain simultaneously. For group nonpainful voices, four individual nonpainful voices were randomly selected and superimposed to simulate neutral voices made simultaneously by a group. Finally, 20 group painful voices and 20 group nonpainful voices were obtained, each lasting 1000 ms and matched for average volumes. The loudness of all voices was set to 70 dB.

**Figure 2.**
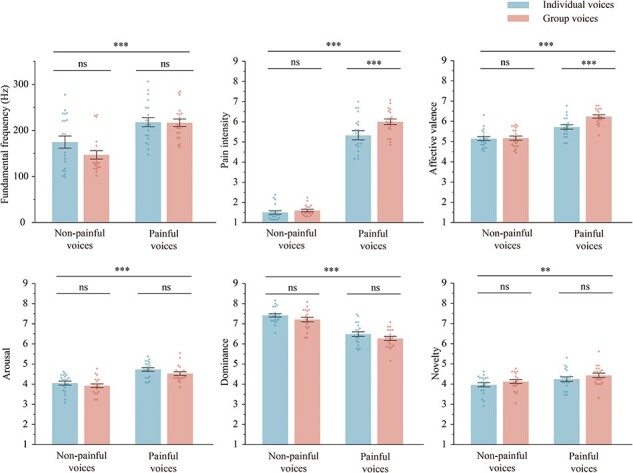
The average fundamental frequency, pain intensity, affective valence, arousal, dominance, and novelty of each type of voices. Results were obtained using two-way ANOVA with the between-participant factors of “stimuli type” (individual voice, group voice) × 2 “pain type” (painful voice, nonpainful voice). Data in the bar charts are expressed as mean ± SEM. Each dot represents the data for one voice. ns: *P* > .05; ** *P* < .01; *** *P* < .001.

We recruited 30 voluntary undergraduate students (15 females; aged 22.13 ± 2.76 years) who did not participate in the formal experiment to assess the pain intensity (1 = “no sensation,” 4 = “pain threshold,” 9 = “unbearable pain”), affective valence (1 = “very happy,” 5 = “neutral,” 9 = “very unhappy”), arousal (1 = “extremely peaceful,” 9 = “extremely excited”), dominance (1 = “extremely out of control,” 9 = “extremely in control”), and novelty (1 = “extremely familiar,” 9 = “extremely unfamiliar”) of each type of voices using the 9-point Likert scales. As shown in Fig. 2, the results of the assessments as well as the average fundamental frequencies of the voices were analyzed with a 2 “stimuli type” (individual voice, group voice) × 2 “pain type” (painful voice, nonpainful voice) two-way repeated-measure ANOVA. All voices used in the experiment and the details of the analysis results are provided in the Supplementary Tables S1 and S2.

### Measurement of trait empathy

The trait cognitive empathy and trait affective empathy of participants were measured using the Questionnaire of Cognitive and Affective Empathy (Reniers et al. 2011, Liang et al. 2019). This scale consisted of 31 items, scored using a 4-point Likert scale (1 = “strongly disagree,” 2 = “slightly disagree,” 3 = “slightly agree,” 4 = “strongly agree”). The scale comprises five dimensions: perspective taking, online simulation, emotional contagion, proximal responsivity, and peripheral responsivity. The perspective taking and online simulation dimensions formed the trait cognitive empathy, while emotional contagion, proximal responsivity, and peripheral responsivity dimensions constituted the trait affective empathy. The Chinese version of the Questionnaire of Cognitive and Affective Empathy demonstrates good reliability and validity (Liang et al. 2019).

### Procedure

The participants conducted the experiment in a quiet room with an ambient temperature of ∼22℃. Stimuli presentation and behavioral data recording were controlled using E-Prime (3.0) program (Psychology Software Tools, Inc, Pittsburgh, PA, USA), while simultaneously recording electroencephalography (EEG) data. Prior to the experiment, all participants completed the Questionnaire of Cognitive and Affective Empathy. The experimental procedure is illustrated in Fig. 1b.

At the beginning of each trial, a voice stimulus lasting for 1000 ms was presented. After hearing the voice, participants were required to quickly and accurately press either the “1” or “2” key (key pressing was counterbalanced across participants to control for potential order effects) to indicate whether the voice was painful or nonpainful. After the participant’s judgment, a blank screen was presented for 200 ms, followed by the participants rating the pain intensities (1 = “no sensation,” 4 = “pain threshold,” 9 = “unbearable pain”) of the voice stimuli and their emotional reactions (1 = “very happy,” 5 = “neutral,” 9 = “very unhappy”) using 9-point Likert scales. The interval between two trials was randomly varied between 1000 ms and 2000 ms.

The present study included four conditions (group painful voice, group nonpainful voice, individual painful voice, and individual nonpainful voice). In one previous study on auditory empathy for pain, each condition included 30 voice stimuli (Liu et al. 2019). To ensure sufficient trials for reducing EEG noise, we included 40 voice stimuli per condition in our study. Each condition had 20 different voice segments as stimuli, each voice stimulus presented twice. Thus, 40 voice stimuli were included in each condition. This necessitated repeating each voice stimulus twice. We used a pseudo-random order for the presentation of the voices to ensure that the same voice did not appear consecutively.

### EEG recording and data analyses

Using the EEG recording system from Brain Products GmbH, EEG data of participants were recorded using a 32-electrode cap installed on the actiCHamp system (Brain Vision LLC, Morrisville, NC, USA), with electrode placement following the international 10–20 system extension. During recording, the reference electrode was placed at FCz, and the ground electrode was placed at the forehead ground site. EEG activity was acquired using DC recording, with a bandwidth ranging from DC to 280 Hz and a continuous sampling rate of 1000 Hz. Impedance between electrodes and the scalp was maintained <5 kΩ.

Using MATLAB R2016a (MathWorks, Natick, MA, USA) and the EEGLAB toolbox for preprocessing and analysis of EEG data. During analysis, the average of the mastoids on both sides was used as the reference, with a bandpass filter ranging from 0.1 Hz to 30 Hz applied. The analysis epoch was from 200 ms before stimulus presentation to 1000 ms after presentation, with the 200 ms period before stimulus presentation serving as the baseline. Artifact removal for eye movements was performed using independent component analysis (ICA) algorithm. After confirming the scalp topography of individual and grand-average ERP waveforms, and referencing previous studies (Liu et al. 2019, Meng et al. 2019a, 2020, Shao et al. 2024), the main ERP components were identified as follows: N1 (Fz, Cz, FC1, and FC2; 90–150 ms), P2 (Fz, Cz, FC1, and FC2; 180–240 ms), and LNC (Fz, F3, F4, FC1, and FC2; 400–800 ms).

### Statistical analyses

Statistical analysis was conducted using MATLAB R2016a (MathWorks, Natick, MA, USA). First, repeated measures ANOVA of 2 “stimuli type” (individual voice, group voice) × 2 “pain type” (painful voice and nonpainful voice) were performed on behavioral data (reaction time, accuracy, pain intensity, and emotional response) and ERP data (N1, P2, and LNC amplitudes). If the interactions between the two factors were significant, simple effects analysis between the two stimuli types were performed for each pain type.

Furthermore, to mitigate the influence of differences in physical properties (such as frequency and timbre) between group and individual voices on the ERP results, the present study also calculated differential ERP amplitudes between painful and nonpainful voices for both group and individual voices. This was done to characterize the psychological components of empathy for pain (Meng et al. 2012, Cui et al. 2016) for both group and individual voices (e.g. differential ERP amplitudes of P2 between painful and nonpainful voices: P2_painful–nonpainful_), and paired-sample *t*-tests were employed to compare the differential ERP amplitudes between group and individual voices.

Finally, Pearson product–moment correlation analysis was conducted to explore the relationship between trait empathy (trait affective empathy and trait cognitive empathy) and ERP differential amplitudes. Pairwise comparisons were corrected using the false discovery rate (FDR) method (Benjamini and Hochberg 1995).

## Results

### Behavioral results

#### Accuracy

The main effect of “stimuli type” was significant [*F*_(1, 33)_ = 11.00, *P* = .002, η^2^_p_ = 0.25], participants showed higher accuracy in distinguishing group voices (0.98 ± 0.02) than individual voices (0.97 ± 0.02).

#### Reaction time

The main effect of “stimuli type” was significant [*F*_(1, 33)_ = 5.22, *P* = .029, η^2^_p_ = 0.14], participants responded faster to group voices (995.09 ± 224.79 ms) than individual voices (1021.59 ± 208.70 ms).

#### Pain intensity

The main effect of “stimuli type” was significant [*F*_(1, 33)_ = 85.98, *P* < .001, η^2^_p_ = 0.72], the pain intensity ratings of group voices (4.32 ± 0.69) were higher than that of individual voices (3.87 ± 0.60). The main effect of “pain type” was significant [*F*_(1, 33)_ = 655.19, *P* < .001, η^2^_p_ = 0.95], the pain intensity ratings of painful voices (6.70 ± 0.98) were higher than nonpainful voices (1.49 ± 0.74). The interaction effect between “stimuli type” and “pain type” was significant [*F*_(1, 33)_ = 76.87, *P* < .001, η^2^_p_ = 0.70]. Simple effect analysis revealed that pain intensity ratings of group painful voices (7.14 ± 1.08) was higher than individual painful voices (6.26 ± 0.94; *P* < .001), whereas the difference between group (1.47 ± 0.77) and individual (1.50 ± 0.73) nonpainful voices was not significant (*P* = .557).

#### Emotional reaction

The main effect of “stimuli type” was significant [*F*_(1, 33)_ = 34.91, *P* < .001, η^2^_p_ = 0.51], participants exhibited more negative emotional reactions to group voices (4.98 ± 0.73) than individual voices (4.78 ± 0.69). The main effect of “pain type” was significant [*F*_(1, 33)_ = 36.65, *P* < .001, η^2^_p_ = 0.53], participants reacted more negatively to painful voices (5.68 ± 1.06) than to nonpainful voices (4.07 ± 1.03). The interaction effect between “stimuli type” and “pain type” was significant [*F*_(1, 33)_ = 15.02, *P* < .001, η^2^_p_ = 0.31]. Simple effect analysis revealed that participants exhibited more negative emotional reactions to group painful voices (5.86 ± 1.21) than individual painful voices (5.51 ± 0.93; *P* < .001), whereas the difference between group and individual nonpainful voices was not significant (4.05 ± 1.05 versus 4.09 ± 0.93; *P* = .263).

No other significant main effect or interaction was found (all *P’*s > .05). More details of the behavioral results see Supplementary Tables S3 and S4.

### ERP data

**Figure 3.**
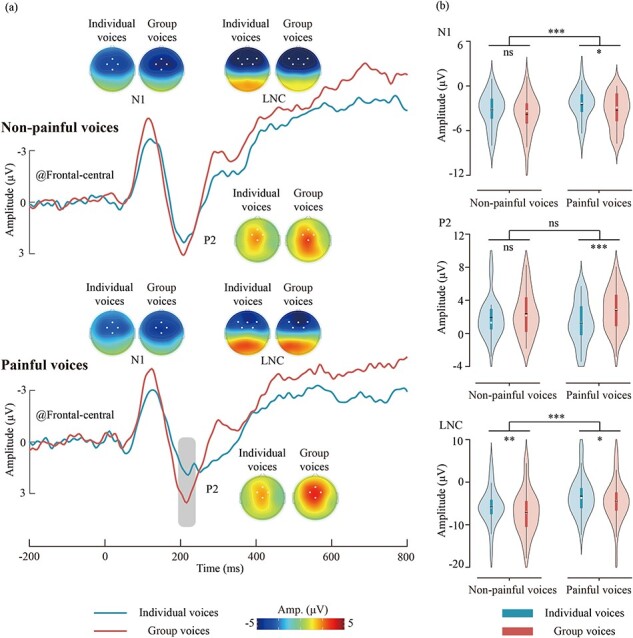
ERP results for different types of voices. (a) ERP waveforms and scalp topography distributions elicited by different types of voices. The dots in the scalp topographies indicate the electrodes included in the analysis. (b) Violin plots depict the N1, P2, and LNC amplitudes. Box plots illustrate the quartile range of the data, with the dot representing the median. ns: *P* > .05, *P < .05, **P < .01, ***P < .001.

ERP waveforms, scalp topographies, and violin plots for the ERP data are depicted in Fig. 3. Results of the statistical analyses of the ERP amplitudes are summarized in Table 1.

**Table 1.** ERP data statistical analysis results

	N1			P2			LNC		
Variable	*F*	*P*	η^2^_p_	*F*	*P*	η^2^_p_	*F*	*P*	η^2^_p_
Stimuli type	**7.08**	**.012**	**0.18**	**19.19**	**<.001**	**0.37**	**18.19**	**<.001**	**0.36**
Pain type	**6.82**	**.013**	**0.17**	0.09	.772	< 0.01	**38.70**	**<.001**	**0.54**
Stimuli type × Pain type	0.35	.558	0.01	**4.72**	**.037**	**0.13**	<0.01	.969	<0.01

Note: The results were obtained through repeated measures analysis of variance, including “Stimuli type” (individual voices, group voices) and “pain type” (painful voices, nonpainful voices). Significant effects (*P* < .05) are highlighted in bold.

#### N1

The main effect of “stimuli type” was significant [*F*_(1, 33)_ = 7.08, *P* = .012, η^2^_p_ = 0.18], group voices (–3.60 ± 2.33 μV) elicited larger N1 amplitudes than individual voices (–2.89 ± 1.99 μV). The main effect of “pain type” was significant [*F*_(1, 33)_ = 6.82, *P* = .013, η^2^_p_ = 0.17], painful voices (–2.94 ± 2.00 μV) elicited smaller N1 amplitudes than nonpainful voices (–3.55 ± 2.25 μV).

#### P2

The main effect of “stimuli type” was significant [*F*_(1, 33)_ = 19.19, *P* < .001, η^2^_p_ = 0.37], group voices (2.57 ± 2.45 μV) elicited larger P2 amplitudes than individual voices (1.58 ± 2.18 μV). The interaction effect between “stimuli type” and “pain type” was significant [*F*_(1, 33)_ = 4.72, *P* = .037, η^2^_p_ = 0.13]. Simple effect analysis revealed that group painful voices (2.77 ± 2.52 μV) elicited larger P2 amplitudes than individual painful voices (1.29 ± 2.58 μV; *P* < .001), whereas the difference between individual and group nonpainful voices was not significant (1.88 ± 2.46 μV versus 2.36 ± 2.70 μV; *P* = .138).

#### LNC

The main effect of “stimuli type” was significant [*F*_(1, 33)_ = 18.19, *P* < .001, η^2^_p_ = 0.36], group voices (–5.79 ± 4.79 μV) elicited larger LNC amplitudes than individual voices (–4.61 ± 4.19 μV). The main effect of “pain type” was significant [*F*_(1, 33)_ = 38.70, *P* < .001, η^2^_p_ = 0.54], painful voices (–3.98 ± 4.49 μV) elicited smaller LNC amplitudes than nonpainful voices (–6.42 ± 4.65 μV).

**Figure 4.**
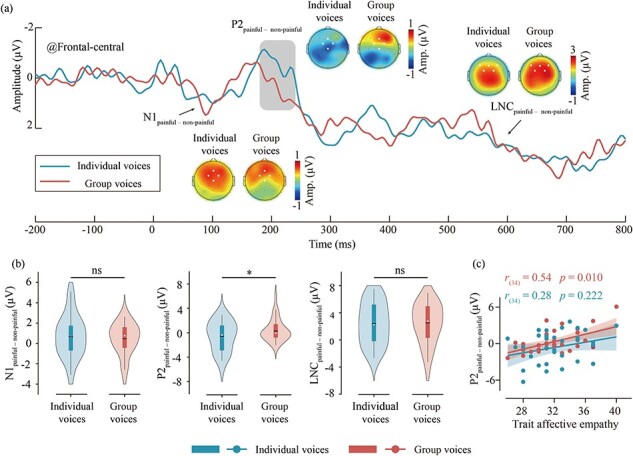
The differential ERP waveforms of group and individual voices and their correlation with trait affective empathy. (a) Waveform and topographical maps of differential ERP of group (red) and individual (green) voices. The regions marked by dots in the topographical maps indicate the electrode points included in the analysis. (b) Results of difference tests of the differential ERP amplitudes between group and individual voices. Violin plots illustrate the distribution of the data, while box plots depict the quartile range of the data, with dots representing the median. ns: *P* > .05, *P < .05. (c) The correlation between trait affective empathy scores and the P2_painful–nonpainful_ amplitudes for group and individual voices. Dots represent the mean values for each participant for group and individual voices, while the lines indicate the best linear fit for each, with the shaded area representing the 95% confidence interval.

Differential ERP waveforms and scalp topography distributions are presented in Fig. 4a, and the violin plots of differential ERP amplitudes are shown in Fig. 4b. P2_painful—nonpainful_ amplitudes of group voices (0.41 ± 1.83 μV) are larger than individual voices [–0.5 ± 2.53 μV; *t*_(33)_ = 2.17, *P* = .037, Cohen’s *d* = 0.37]. No other significant main effect or interaction was found (all *P’*s > .05). More details of the ERP results see Supplementary Table S5.

### The relationship between trait empathy and differential ERP amplitude

The trait affective empathy scores of participants were positively correlated with the P2_painful–nonpainful_ amplitudes for group voices [*r*_(*34*)_ = 0.54, *P *= .010], as depicted in Fig. 4c. However, there was no significant correlation between the trait affective empathy scores and the P2_painful–nonpainful_ amplitudes for individual voices [*r*_(*34*)_ = 0.28, *P* = .222]. In addition, the trait cognitive empathy scores did not show significant correlation with the differential ERP amplitudes (all *P’s* > .05).

## Discussion

The present study utilized group painful voices and individual painful voices to explore the characteristics and cognitive neural mechanisms of group empathy for pain through behavioral and ERP techniques. The findings indicated that higher pain intensity ratings, more negative emotional responses, and larger P2 amplitudes were responded to group painful voices than to individual painful voices. These results imply that people are inclined to empathize more with group’s pain than with individual’s pain. This series of findings confirmed the presence of empathy for pain towards group’s suffering and suggested that group empathy for pain may be stronger than individual empathy for pain.

Consistent with prior literature of empathy for pain in the auditory modality (Liu et al. 2019, Meng et al. 2019a, 2019b, 2020), the present study revealed that, in comparison to nonpainful voices, participants exhibited higher pain intensity ratings, more negative emotional responses and smaller N1 and LNC components to painful voices. Given that N1 reflects early sensory processing (Choi et al. 2014), and LNC represents late cognitive empathy (Meng et al. 2019a, 2019b, Shao et al. 2024) in auditory empathy for pain, participants may engage in facilitated early sensory processing and late cognitive evaluation when exposed to others’ painful voices. However, the larger ERP amplitudes for nonpainful voices may also be attributed to the lower frequency than painful voices (Huang et al. 2006, Zhang et al. 2023).

Previous studies have shown that, in comparison with when the target face’s emotions were different from the surrounding faces, the target face’s emotions were recognized more quickly and accurately when the target face’s emotions were congruent with the surrounding faces (Mumenthaler and Sander 2012, Mumenthaler et al. 2018, Wang et al. 2024). Our results supported the previous study, showing that compared to individual voices, quicker and more accurate responses, higher pain intensity ratings, and more negative emotional responses, as well as larger N1, P2, and LNC amplitudes were made to judge group voices. These results suggest that group information may facilitate empathetic processing (Haberman and Whitney 2007, Li et al. 2016), possibly due to the conspicuous survival importance of group-related cues (Smith et al. 2007).

Interestingly, the present study found that participants report higher pain intensity ratings, more negative emotional responses, and larger P2 amplitudes for group painful voices than individual painful voices. But no difference was found between group and individual nonpainful voices. These results were consistent with the previous study, which indicated that group painful faces elicited larger P2 amplitudes than individual painful faces (Mei et al. 2024). Considering that the P2 component represents the automatic emotional contagion and affective sharing processes of empathy for pain in the auditory modality (Choi et al. 2014, Meng et al. 2019a), group painful voices may play a role in facilitating the emotional empathetic processes of auditory empathy for pain. However, previous studies have indicated that the P2 component is modulated not only by emotional quality (Paulmann et al. 2009, Yeh et al. 2016) but also by contextual information (van Wassenhove et al. 2005). Therefore, the P2 component may be related not only to the processing of empathy but also to the social perception of group information.

Although the present study has attempted to match the physical properties (including the duration and average loudness) between different types of voices, other physical properties, such as frequency and timbre of auditory stimuli may also influence the ERP waveform. Therefore, in addition to analyzing the raw ERP amplitudes, the present study calculated differential ERP amplitudes between painful and nonpainful stimuli for group and individual voices separately, to reduce the potential impact of stimulus physical properties (Meng et al. 2012, Cui et al. 2016). The statistical results of the differential ERP amplitudes were consistent with those of the raw ERP amplitudes, showing that P2_painful–nonpainful_ amplitudes were larger for group voices than individual voices. In addition, the trait affective empathy scores were significantly positively correlated with the P2_painful–nonpainful_ amplitudes for group voices, indicating that the stronger the participants’ trait affective empathy, the larger the P2_painful–nonpainful_ amplitudes observed. Previous research indicates that individuals with higher trait affective empathy are more sensitive to emotional information (Reniers et al. 2011, Liang et al. 2019). In addition, the effect of group stimuli on empathy also occurs at P2 in visual modality, which indicated that group stimuli involved more emotional social information than individual stimuli (Mei et al. 2024, Wang et al. 2024). Therefore, the positive correlation between trait affective empathy and the P2_painful–nonpainful_ component may indicate that group empathy for pain may involve more emotional social information in auditory modality. These results suggested that compared to individual empathy for pain, affective empathy in group empathy for pain may be enhanced.

The above results possibly because humans are social beings, capable of experiencing emotions that align with their group identity (Smith et al. 2007). Humans tend to employ ensemble encoding when faced with group emotions, integrating and averaging information from all individuals to recognize emotions (Haberman and Whitney 2007). When the target stimuli are ambiguous, participants are more inclined to rely on contextual information from the group to judge emotions (Leleu et al. 2015). Compared to individual stimuli, group stimuli not only contain more emotional information but also imply more social interaction-related group information (Mei et al. 2024, Wang et al. 2024), making it easier for participants to empathize with group painful voices than individual painful voices.

It is worth noting that the present study has some limitations. First, since this study was limited to healthy adults, it remains to be verified whether the results of this study can be extrapolated to the children. Second, the group voices in the present study were made by superimposing four individual voices, thus the effect of group voices with a larger number of individual voices was needed to be explored. Third, compared to nonpainful voices, painful voices elicited smaller ERP amplitudes. This may be due to the higher frequency of painful voices compared to nonpainful voices. Interpretation of this result requires caution. Future research on auditory empathy for pain should pay particular attention to whether the physical attributes of painful and nonpainful voices are matched. Fourth, no differences were found between painful and nonpainful voices in the P2 component. Therefore, any inferences regarding the P2 component in auditory modality as an indicator of empathy for pain should be made with caution. Lastly, the present study only explored group empathy for pain, and future research should explore whether similar group facilitate effects exist for other emotions.

## Conclusion

The present study employed behavioral and ERP techniques to investigate group empathy for pain in the auditory modality. The results indicated that participants reported stronger empathetic responses to group painful voices than individual painful voices, suggesting that group empathy for pain may facilitate affective empathetic processing, at least in the auditory modality. This study provided initial insights into the group facilitate effects of group empathy for pain.

## Supplementary Material

nsae074_Supp

## Data Availability

The data and experimental materials related to this article can be found at the following link: https://pan.baidu.com/s/1sdRE6-XQ2pnOScwp5FoEeA?pwd=xoto.
