# Supplementary material for: Group empathy for pain is stronger than individual empathy for pain in the auditory modality
Source: Soc Cogn Affect Neurosci. 2024 Oct 17;19(1):nsae074. doi: 10.1093/scan/nsae074 (PMC11523625; doi:10.1093/scan/nsae074)
Supplement: nsae074_Supp [file nsae074_supp.zip › nsae074_Supp/scan-24-145-File007.docx]

**Table S1** Descriptive statistics for assessments and fundamental frequencies of the voices

|  | Individual non-painful voices (*n* = 20) | | Group non-painful voices (*n* = 20) | | Individual painful voices (*n* = 20) | | Group painful voices (*n* = 20) | |
| --- | --- | --- | --- | --- | --- | --- | --- | --- |
|  | *Mean* | *SD* | *Mean* | *SD* | *Mean* | *SD* | *Mean* | *SD* |
| Fundamental frequencies | 174.69 | 58.85 | 146.92 | 40.92 | 217.88 | 44.19 | 216.64 | 36.51 |
| Pain intensity | 1.50 | 0.38 | 1.60 | 0.26 | 5.54 | 0.84 | 6.23 | 0.50 |
| Affective valence | 5.14 | 0.43 | 5.17 | 0.46 | 5.72 | 0.49 | 6.24 | 0.37 |
| Arousal | 4.05 | 0.44 | 3.92 | 0.41 | 4.73 | 0.40 | 4.53 | 0.41 |
| Dominance | 7.42 | 0.39 | 7.21 | 0.50 | 6.48 | 0.52 | 6.27 | 0.47 |
| Novelty | 3.96 | 0.46 | 4.13 | 0.43 | 4.25 | 0.50 | 4.43 | 0.49 |
