# Supplementary material for: Group empathy for pain is stronger than individual empathy for pain in the auditory modality
Source: Soc Cogn Affect Neurosci. 2024 Oct 17;19(1):nsae074. doi: 10.1093/scan/nsae074 (PMC11523625; doi:10.1093/scan/nsae074)
Supplement: nsae074_Supp [file nsae074_supp.zip › nsae074_Supp/scan-24-145-File008.docx]

**Table S2** The statistical analysis results of assessments and fundamental frequencies of the voices

|  | Stimuli type | | | Pain type | | | Stimuli type × Pain type | | |
| --- | --- | --- | --- | --- | --- | --- | --- | --- | --- |
|  | *F* | *p* | η^2^_p_ | *F* | *p* | η^2^_p_ | *F* | *p* | η^2^_p_ |
| Fundamental frequencies | 2.00 | 0.161 | 0.03 | **30.27** | **< 0.001** | **0.29** | 1.67 | 0.200 | 0.02 |
| Pain intensity | **10.61** | **0.002** | **0.12** | **1294.99** | **< 0.001** | **0.95** | **6.12** | **0.016** | **0.08** |
| Affective valence | **7.72** | **0.007** | **0.09** | **69.73** | **< 0.001** | **0.48** | **6.30** | **0.014** | **0.08** |
| Arousal | 3.22 | 0.077 | 0.04 | **47.80** | **< 0.001** | **0.39** | 0.15 | 0.697 | < 0.01 |
| Dominance | **4.00** | **0.049** | **0.05** | **78.40** | **< 0.001** | **0.51** | < 0.01 | 0.985 | < 0.01 |
| Novelty | 2.77 | 0.100 | 0.04 | **7.88** | **0.006** | **0.09** | 0.01 | 0.925 | < 0.01 |
