# Supplementary material for: Group empathy for pain is stronger than individual empathy for pain in the auditory modality
Source: Soc Cogn Affect Neurosci. 2024 Oct 17;19(1):nsae074. doi: 10.1093/scan/nsae074 (PMC11523625; doi:10.1093/scan/nsae074)
Supplement: nsae074_Supp [file nsae074_supp.zip › nsae074_Supp/scan-24-145-File009.docx]

**Table S3** Descriptive statistics for behavioral data

|  | Accuracy | | Reaction time (ms) | | Pain intensity | | Emotional reaction | |
| --- | --- | --- | --- | --- | --- | --- | --- | --- |
|  | *Mean* | *SD* | *Mean* | *SD* | *Mean* | *SD* | *Mean* | *SD* |
| Individual non-painful voices | 0.97 | 0.03 | 1022.36 | 219.68 | 1.47 | 0.73 | 4.05 | 1.05 |
| Individual painful voices | 0.96 | 0.04 | 1020.82 | 212.82 | 6.26 | 0.94 | 5.51 | 0.93 |
| Group non-painful voices | 0.98 | 0.02 | 1002.99 | 238.20 | 1.50 | 0.76 | 4.09 | 1.03 |
| Group painful voices | 0.97 | 0.04 | 987.19 | 224.55 | 7.14 | 1.08 | 5.86 | 1.21 |
