# Supplementary material for: Group empathy for pain is stronger than individual empathy for pain in the auditory modality
Source: Soc Cogn Affect Neurosci. 2024 Oct 17;19(1):nsae074. doi: 10.1093/scan/nsae074 (PMC11523625; doi:10.1093/scan/nsae074)
Supplement: nsae074_Supp [file nsae074_supp.zip › nsae074_Supp/scan-24-145-File010.docx]

**Table S4** Behavioral data statistical analysis results

|  | Accuracy | | | Reaction time | | | Pain intensity | | | Emotional reaction | | |
| --- | --- | --- | --- | --- | --- | --- | --- | --- | --- | --- | --- | --- |
|  | *F* | *p* | η^2^_p_ | *F* | *p* | η^2^_p_ | *F* | *p* | η^2^_p_ | *F* | *p* | η^2^_p_ |
| Stimuli type | **11.00** | **0.002** | **0.25** | **5.22** | **0.029** | **0.137** | **85.98** | **< 0.001** | **0.72** | **34.91** | **< 0.001** | **0.51** |
| Pain type | 3.02 | 0.093 | 0.84 | 0.24 | 0.626 | 0.01 | **655.19** | **< 0.001** | **0.95** | **36.66** | **< 0.001** | **0.53** |
| Stimuli type × Pain type | 0.97 | 0.332 | 0.03 | 0.88 | 0.356 | 0.03 | **76.87** | **< 0.001** | **0.70** | **15.02** | **< 0.001** | **0.31** |
