# Supplementary material for: Group empathy for pain is stronger than individual empathy for pain in the auditory modality
Source: Soc Cogn Affect Neurosci. 2024 Oct 17;19(1):nsae074. doi: 10.1093/scan/nsae074 (PMC11523625; doi:10.1093/scan/nsae074)
Supplement: nsae074_Supp [file nsae074_supp.zip › nsae074_Supp/scan-24-145-File011.docx]

**Table S5** The differential ERP waveforms (painful – non-painful) between group and individual voices

|  | Individual voices | | Group voices | | *t*_(33)_ | *p* | Cohen’s *d* |
| --- | --- | --- | --- | --- | --- | --- | --- |
|  | *Mean* | *SD* | *Mean* | *SD* |  |  |  |
| N1_painful – non-painful_ | 0.73 | 2.10 | 0.47 | 1.57 | 0.59 | 0.558 | 0.10 |
| P2_painful – non-painful_ | -0.58 | 2.53 | 0.41 | 1.83 | **2.17** | **0.037** | **0.37** |
| LNC_painful – non-painful_ | 2.42 | 3.16 | 2.45 | 3.04 | 0.04 | 0.969 | 0.01 |
